# Biliary Colic

**Published:** 1900-04-21

**Authors:** 


### BILIARY COLIC.

Dr. Keay,[1] of Greenwich, makes a suggestion as to the utility of chloroform in arriving at a diagnosis in cases of biliary colic. So far as pain is to be taken as a guide the matter is apt to be complicated in these cases by the close proximity and intimate connection between such important organs as the stomach, duodenum, pancreas, liver, gall-bladder, and bile ducts, and by the fact that these organs can for the most part be only indirectly explored. Dr. Keay has come to the conclusion that the seat of the pain which precedes the passage of gall-stones has seldom, if ever, been correctly described in text books, and he insists more especially upon the fact that it is first felt in the back. Long before an acute attack there are generally pains in the back, often mistaken for lumbago or for some spinal mischief; but when a calculus is fairly lodged and trying to find its way through the bile ducts, the pain, he says, begins about the tenth or eleventh dorsal vertebræ, gradually passes round, and, though not quite disappearing, gives place to excruciating pain in the right, and often in the left hypochondrium, middle or lower abdomen, frequently above the left nipple, but practically never the right shoulder. Pain often begins, it is admitted, in the right hypochondrium, but it is held that such pain is due to irritation in the gall-bladder, and is not followed by the passage of a gall-stone. When a patient is suffering from biliary colic the pain may be shooting in all directions, and may be so exquisite that little can be learned regarding it. But a little chloroform, not enough to produce unconsciousness, will gradually cause the distant or referred pain to cease until there remains only a distinctly localised pain at the bile ducts.

[1] Brit. Med Jour., April 14.

